# Gender and other intersecting factors in antimicrobial resistance for infectious diseases of poverty: a systematic evidence gap analysis in low- and lower-middle-income countries

**DOI:** 10.1186/s12889-026-26990-5

**Published:** 2026-03-16

**Authors:** Dhanajayan Govindan, Yuvaraj Krishnamoorthy, Marie Gilbert Majella, Monica Karunakaran

**Affiliations:** 1 Evidence Synthesis Unit, PROPUL Evidence LLP, Chennai, 600099 India; 2 Epidemiology and Public Health Unit, PROPUL Evidence LLP, Chennai, 600099 India; 3 PROPUL Evidence LLP, Chennai, India

**Keywords:** Antimicrobial Resistance, Gender, Infectious Diseases, Intersectionality

## Abstract

**Background:**

Gender influences health outcomes by affecting exposure to risk factors, healthcare access, and health-seeking behaviours. Yet, many studies fail to consider how these gendered experiences interact with other social factors, such as age, socioeconomic status, and ethnicity. Our study systematically mapped existing research to identify gaps in understanding how these factors affect service delivery outcomes related to antimicrobial resistance (AMR) for infectious diseases of poverty.

**Methods:**

We conducted a systematic review and evidence gap map analysis following PRISMA 2020 guidelines. A comprehensive search was conducted across five major databases (MEDLINE, EMBASE, Scopus, Cochrane Library, and Web of Science) for studies published up to March 31, 2024. To synthesise the evidence, we developed and applied a specialized ‘Guide for an Intersectionality Approach’, adapted from established frameworks, to evaluate how gender identity, gender roles, and norms intersect with social factors like age, socioeconomic status, and ethnicity to influence AMR service delivery outcomes in Low- and Lower-Middle-Income Countries (LLMICs) for malaria, tuberculosis (TB) and neglected tropical diseases (NTDs).

**Results:**

Out of 38,310 identified records, 14 studies met the inclusion criteria, all of which focused exclusively on TB; no eligible evidence was found for other infectious diseases of poverty like malaria or NTDs. While all included studies provided sex-disaggregated data, there was a significant lack of deeper intersectional analysis, with zero studies exploring non-traditional gender roles, power dynamics, or the compounded effects of intersecting social stratifiers. Findings indicated sex parity in diagnostic access but revealed male-specific vulnerabilities in treatment retention and a 4.06-fold higher risk of mortality for men. Conversely, women faced higher risks of treatment delays in certain contexts and often relied on informal antibiotic sources.

**Conclusion:**

There is a lack of studies evaluating how social determinants shaped by gender dynamics (roles, norms, relations, and power) influences inequities and vulnerabilities caused by AMR in LMICs. This gap limits our understanding of how these intersections affect antibiotic use, healthcare access, and treatment adherence. Future research should use intersectionality as an analytical framework and mixed-methods approaches to develop more inclusive and equitable health interventions that address the compounded inequities and vulnerabilities associated with gender and other social factors.

**Supplementary Information:**

The online version contains supplementary material available at 10.1186/s12889-026-26990-5.

## Introduction

Infectious diseases of poverty, such as Tuberculosis (TB), malaria, and NTDs like dengue, schistosomiasis, and Chagas disease, disproportionately affect marginalised populations in low income countries (LICs) and low middle income countries (LMICs) [1]. These diseases thrive in conditions of poverty, where there is limited access to healthcare, inadequate sanitation, and poor living conditions [2]. Addressing these diseases requires a comprehensive approach that considers the social factors of health and other vulnerabilities of affected populations. Service delivery-related outcomes, including access to diagnostics, treatment adherence, and quality of care, are crucial components in effectively managing these diseases. Ensuring equitable healthcare services that are accessible, affordable, and culturally appropriate is essential for improving health outcomes and reducing the burden of these diseases [3].

Antimicrobial resistance (AMR) compounds these issues by rendering standard treatments ineffective, making it harder to control and treat infections [4]. Misuse and overuse of antibiotics, often driven by lack of regulation and healthcare access, accelerate the development of resistant strains. This not only complicates treatment but also increases healthcare costs and mortality rates [5, 6]. Marginalised populations in LMICs are disproportionately affected by AMR due to limited access to quality healthcare, lack of awareness, and poor healthcare infrastructure [7]. In these settings, antibiotics are often available over the counter without a prescription, leading to misuse. Additionally, the burden of infectious diseases is higher in these populations, necessitating frequent use of antimicrobials and increasing the risk of developing resistance [8].

Gender identity, gender roles and gender norms play a crucial role in the development of AMR. Women, particularly those who are primary caregivers, are often responsible for managing illness within households, which can influence patterns of antibiotic use and adherence to treatment guidelines. Gender-based differences in healthcare access and health-seeking behaviour affect how men and women are exposed to and manage AMR [9, 10]. Intersectionality can be used as a framework used to explore how different social factors, such as gender, race, or socioeconomic status, intersect and overlap, under complex systems and structures of power, resulting in experiences of discrimination, disadvantage, or privilege [11]. Originating from feminist and critical race theories, this concept offers an in-depth understanding of how various social factors create inequity and vulnerability to diseases and access to healthcare [12]. An intersectionality approach is particularly vital in understanding how gender intersects with other factors like socioeconomic status, ethnicity, and geographical location, and aggravates the health disparities in LMICs.

Given this, it is critical to assess the combined vulnerabilities faced by populations in LMICs and LICs through a comprehensive review, highlighting areas where evidence is lacking. Examining how gender identity, gender roles and norms and AMR impact service delivery for infectious diseases of poverty will help to understand how these factors intersect to worsen health disparities. Hence, this study was conducted to systematically map existing research, data, and interventions related to gender, social factors, and AMR on service delivery outcomes for infectious diseases of poverty within LMICs and LICs using an intersectional gender lens.

## Methods

### Study design

This study is a systematic review and evidence gap map (EGM) conducted in accordance with Preferred Reporting Items for Systematic Reviews and Meta-Analyses (PRISMA) 2020 guidelines [13]. While the review includes existing systematic reviews, its primary purpose is to systematically map the available evidence and identify specific research lacunae regarding intersectional gender analysis in AMR.

### Inclusion criteria

#### Design/type of studies

Peer-reviewed articles, including quantitative, qualitative, and mixed-methods studies, systematic reviews with/without meta-analyses, policy documents, reports and grey literature from reliable sources were eligible.

#### Study population

Studies done amongst populations in LICs and LMICs with AMR to any one of the following disease groups were eligible: Malaria, TB, neglected tropical diseases (NTDs) [14], including dengue, snakebite envenoming, chikungunya, Buruli ulcer, dracunculiasis, Chagas disease, Rabies, foodborne trematodiases, Scabies, Onchocerciasis, Human African trypanosomiasis, Leishmaniasis, Schistosomiasis, Leprosy, Trachoma, Lymphatic filariasis, Echinococcosis, Mycetoma, Soil-transmitted helminthiases, chromoblastomycosis, other deep mycoses, Noma, and other ectoparasitoses, Taeniasis/cysticercosis, and Yaws. Countries were identified as Low-Income (LIC) or Lower-Middle-Income (LMIC) using the World Bank Country and Lending Groups classification for the 2023–2024 fiscal year [15].

#### Exposure

Studies were eligible if they are reporting how gender intersects with any one of the following biological and social factors: Age, Sex, Gender identity, Disability, Ethnicity, Race, Education, Occupation, Socioeconomic status, Income, Residence Characteristics, Caste, Social class, Religion, immigration status, marital status, sexual orientation in AMR context were eligible for inclusion and considered as primary objective. Studies reporting sex-disaggregated outcomes in the context of AMR were also considered eligible for inclusion under the secondary objective.

#### Outcomes

Studies reporting any one of the following service delivery outcomes were eligible: healthcare access, quality of healthcare, adherence to treatment, and utilisation of healthcare services.

### Exclusion criteria

To ensure the thematic focus and methodological rigour of this review, the following exclusion criteria were applied:

1. Studies conducted in countries classified as High-Income or Upper-Middle-Income. Research not focused on the predefined infectious diseases of poverty or where AMR was not a primary outcome.
2. Studies that lacked sex-disaggregated data or failed to report on at least one intersecting social stratifier.
3. Editorials, commentaries, opinion pieces, and conference abstracts lacking peer-reviewed methodological detail or accessible full-text data.

### Information sources

The following information sources were utilised for this review: MEDLINE, EMBASE, Scopus, Cochrane Library and Web of Science. Additionally, Google Scholar was searched for any additional evidence and a grey literature search was also done through searches of institutional websites, conference proceedings, and policy documents.

### Search strategy

Comprehensive search strategies were tailored to each database to maximise the retrieval of relevant studies. The search has used a combination of keywords and MeSH terms related to intersectionality, gender, social factors, AMR, and the eligible infectious diseases in this review. The detailed strategy in each of the databases is provided in the Supplementary File. The time limit of the search had no restriction for the starting point (i.e., inception of each of those databases), and the endpoint of the search was till March 31, 2024.

### Study screening

All records retrieved from the databases were imported into the Rayyan online tool, where duplicate records were identified and removed using the automated feature of the platform. Two independent reviewers (YK and DG) then performed an initial screening of the remaining records by examining the titles and abstracts. Full texts were obtained for studies that appeared relevant based on this initial review. The full texts were subjected to secondary, comprehensive screening by the same reviewers to confirm their alignment with the predefined eligibility criteria. Any disagreements/conflicts during the process were resolved through discussions involving entire project team.

### Data items and extraction process

Two independent investigators (YK and DG) did the data extraction process for each of the retrieved full-text records. The data was extracted using the template incorporating the following data items: General Information: Study ID, Authors, Year of Publication, Journal/Source, Country/Region. Study Characteristics: Publication type, Study Design, Participants, Study Region, Sample Size, Social Factors, NTD/TB/Malaria, AMR, LIC/LMIC, Inclusion/Exclusion Criteria, Study Period, Healthcare Intervention, Public Health Interventions, Service Delivery Component Outcomes: Main outcomes and any other secondary outcomes measured, outcome assessment tool/scale.

Quality Appraisal: The methodological rigour of the included studies was assessed using standardised checklists to ensure the reliability of the evidence mapping. Cross-sectional studies were evaluated using the Appraisal tool for Cross-Sectional Studies (AXIS), while cohort studies were assessed using the Critical Appraisal Skills Programme (CASP) checklist. Each study was categorised as having a Low, Moderate, or High Risk of Bias.

### Data synthesis

#### Development of a tailored intersectionality guide

To synthesise the evidence effectively, a specialised guide called “Guide to approach AMR, Gender, and Health Inequities with an intersectional lens” was developed to capture the complex interactions between gender, social factors, and AMR. The foundational framework of this guide was adapted from the study by Djoudi et al. (2016), which examined gender inequalities in climate change research using an intersectionality approach [16]. To ensure that this guide was relevant to current specific objectives, it underwent a series of refinements, guided by feedback from experts in gender studies and intersectionality, public health, infectious diseases, AMR, and field epidemiology.

#### Guiding questions for study assessment

To assess each study using the intersectionality guide, a set of guiding questions was adapted from Djoudi et al. (2016) [16] and underwent further refinement to fit the context of AMR, based on expert consultations. The operational definitions for this review are as follows:

- *Gender*: “The socially constructed roles, behaviours, activities, attributes and opportunities that any society considers appropriate for men and women, boys and girls, and people with non-binary identities [17].
- *Intersectionality*: Intersectionality can be used as an analytical lens that examines how different social factors (such as gender, class, ‘race’, education, ethnicity, age, geographic location, religion, migration status, ability, disability, sexuality, etc.) overlap and intersect simultaneously to create different experiences of privilege, vulnerability and/or marginalisation [17].
- *Power dynamics*: Power dynamics result from an imbalance between individuals/groups in access to resources. Those with greater economic and social capital hold more power and greater influence. An individual’s ability, race, gender identity, and sexual orientation are often associated with difference instatus, power, and privilege [18].
- *Agency*: The capacity of an individual to actively and independently choose and to affect change; free will or self-determination. It is an expression of autonomy against social institutions, structures, and cultural forces [19].
- *Emancipation*: The concept of emancipation refers to an entity’s liberation from control, dependence, restraint, confinement, restriction, repression, slavery, or domination [20].

*Gap Analysis*: The gap analysis involved a detailed assessment of the studies included, quantifying them based on criteria within the guide to identify areas of focus specifically in the context of AMR. The quantification process revealed areas within the guide that were either underrepresented or overrepresented in the existing literature.

The gap analysis also involved extracting and organising country-specific findings to understand the regional focus within LICs and LMICs. The analysis concentrated on several critical areas, including:

- *Reported Outcomes*: Cataloguing service delivery outcomes, such as access to healthcare, quality of care, and treatment adherence.
- *Study Design*: To ensure methodological rigour during the synthesis of evidence, findings were segregated and synthesised according to the original study designs of the included literature.
- *Explored Social Factors*: Evaluating the extent to which each study considered the role of gender and other social factors such as age, socioeconomic status, ethnicity, and additional social factors within the context of AMR.

## Results

**Fig. 1:**
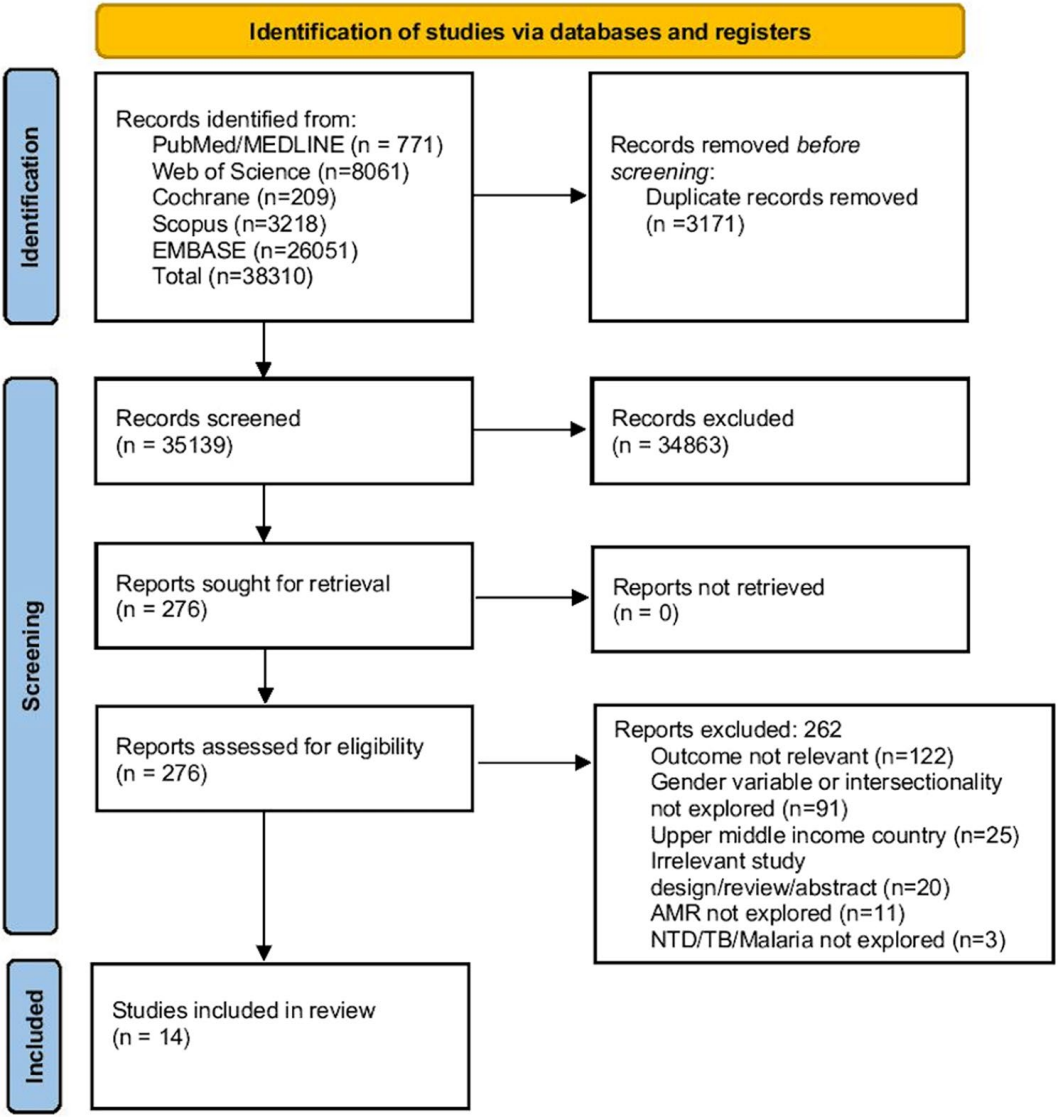
PRISMA Flowchart for the Systematic Evidence Gap Analysis on the Intersection of Gender and Antimicrobial Resistance in Infectious Diseases of Poverty in Low- and Lower-Middle-Income Countries. Description: This PRISMA flowchart outlines the systematic process of identifying, screening, and selecting studies for inclusion in the review. It details the number of records identified from various databases, the number of duplicates removed, and the number of articles excluded at each stage of the screening process, with reasons for exclusion, to arrive at the final number of included studies

Overall, 38,310 articles were identified across multiple databases. After the removal of duplicates and primary screening, 34,863 articles were excluded for not meeting the initial eligibility criteria. A further 276 articles were screened for detailed eligibility, of which 262 were excluded. Finally, 14 articles were included in the systematic review and evidence gap map (Fig. 1) [21–34]. None of these 14 articles met the primary objective-related criteria regarding deep intersectional analysis; however, all satisfied the secondary objective-related criteria for sex-disaggregated reporting.

### Characteristics of the included studies

Although our search strategy encompassed wide range of infectious diseases of poverty, including Malaria and NTDs, only studies focusing on TB met the final inclusion criteria. This extreme concentration of evidence suggests a significant research gap regarding intersectional and gender analysis in AMR for all other infectious diseases of poverty.

Table 1 includes 14 studies with five from India, two from Myanmar and one each from Nigeria, Zimbabwe, Papua New Guinea, Uganda, Cameroon, Pakistan and Kyrgyz. Sample sizes across the studies varied from 100 to over 42,000 participants. The primary outcomes assessed in these TB-focused studies involved the burden of AMR on healthcare systems, service delivery efficiency, and delays in diagnosis and treatment [21–34]. 

**Table 1:** Characteristics of the included studies (*N* = 14)

Author	Country	Study Design	Participants	Sample size	Disease	Anti-microbial drug	Social factor	Outcome
Oga-Omenka et al. 2019 [21]	Nigeria	Retrospective cohort study	Patients diagnosed with Mycobacterium tuberculosis and resistance to rifampicin	996	Tuberculosis	Rifampicin	Age, sex, geographical zone, Urban/rural	Treatment initiation rates and timeliness of treatment
Timire et al. 2019 [22]	Zimbabwe	Cohort study using secondary data	Pulmonary TB patients diagnosed with RR-TB on the Xpert MTB/RIF	133	Tuberculosis	Rifampicin	Age, sex, Residence	Access to diagnosis testing.
Kumar et al. 2024 [23]	India	Prospective observational study	All smear and culture positive pulmonary tuberculosis patients (age ≥ 14 years)	171	Tuberculosis	Isoniazid and rifampicin	Age, sex, Education, Socioeconomic status.	Treatment interruption pattern
Hapolo et al. 2019 [24]	Papua New Guinea	Retrospective cohort study	Patients on TB treatment	360	Tuberculosis	MDR	Age, sex	Treatment delay and initiation, Risk factor for delayed treatment
Htun et al. 2018 [25]	Myanmar	Retrospective study	Adult TB patients who had registered and started treatment with the standard regimen	210	Tuberculosis	Rifampicin	Age, sex	Treatment delay, disease severity, treatment adherence, treatment outcomes.
Izudi et al. 2020 [26]	Uganda	Cross-sectional study	Previously treated patients with bacteriologically confirmed pulmonary tuberculosis	135	Tuberculosis	MDR	Age, sex	Treatment success rate, MDR/RR TB surveillance rates
Jouego et al. 2022 [27]	Cameroon	Retrospective cohort study	Patients diagnosed with Rr-TB on Xpert MTB/RIF	871	Tuberculosis	Rifampicin	Age, sex	Treatment outcome
Shewade et al. 2017 [28]	India	Retrospective cohort study	Presumptive MDR-TB patients who underwent genotypic DST	628	Tuberculosis	MDR	Age, sex	Treatment attrition rates
Shewade et al. 2018 [29]	India	Retrospective cohort study	Presumptive MDR-TB patients who underwent genotypic DST	2212	Tuberculosis	MDR	Age, sex	Delay in diagnostic testing
Arif et al. 2021 [30]	Pakistan	Cohort Study	Newly diagnosed patients with MDR-TB with age 12 and above.	438	Tuberculosis	MDR	Age, sex, Income, Education	Treatment outcome, survival rate of MDR-TB patients
Kyrbashov et al. 2023 [31]	Kyrgyz	Cohort study	Patients who started second-line drug regimens for RR/MDR-TB and XDR treatment	535	Tuberculosis	MDR	Age, sex, Region, Migrant status, Economic status	Diagnosis and treatment initiation, Treatment outcome.
Raizada et al. 2015 [32]	India	Prospective demonstration study	HIV-infected presumptive pulmonary TB cases across 18 sub-district tuberculosis units in India	2787	Tuberculosis	Rifampicin	Age, sex, Geographical location	Tb diagnosis accuracy and rifampicin resistance detection
Raizada et al. 2018 [33]	India	Observational implementation study	Presumptive pediatric TB cases (0–14 years old)	42,238	Tuberculosis	Rifampicin	Age, sex, Health care facilities	TB diagnosis rates, rifampicin resistance level
Sidharta et al. 2018 [34]	Myanmar	Cross–sectional study	Newly diagnosed adult pulmonary DR-TB patients with confirmed rifampicin resistance.	202	Tuberculosis	NA	Age, sex, Income, Ethnicity	Treatment access, Health care behaviour

### Gap analysis

The quantification process, summarized in Table 2, revealed a profound evidence vacuum; out of 14 studies, zero explored how traditional or non-traditional gender roles influence AMR vulnerability, and none (0/14) investigated power dynamics or individual agency. This reveals that while sex-disaggregated data is being collected, the ‘intersectional’ component of the research is entirely absent in current LLMIC literature.

**Table 2:** Results of evidence gap analysis utilising final intersectionality guide

Guide for Evidence Gap Map Analysis	No. of studies
1. General Overview:	
Total number of studies included in the evidence gap map	14
Summarise based on publication year range, geographic focus, and study methodologies.	-
2. Gender-relevant Aspects:	
Number of Studies Exploring traditional and non-traditional gender roles in relation to vulnerability	0
Number of Studies Exploring traditional and non-traditional gender roles in relation to adaptive capacity	0
Number of Studies Exploring differences in exposure to AMR impacts based on gender	0
Number of Studies Exploring adaptive strategies that are tailored to specific genders	0
Number of Studies Exploring shifts in societal gender norms and practices in response to AMR impacts	0
3. Intersectionality with social factors:	
Number of Studies considering age as a variable in addition to gender in the analysis	0
Number of Studies considering ethnicity as a variable in addition to gender in the analysis	0
Number of Studies considering occupation as a variable in addition to gender in the analysis	0
Number of Studies considering wealth as a variable in addition to gender in the analysis	0
Number of Studies considering mobility as a variable in addition to gender in the analysis	0
Number of Studies considering homelessness as a variable in addition to gender in the analysis	0
Number of Studies considering education/health literacy as a variable in addition to gender in the analysis	0
Number of Studies considering geographic location as a variable in addition to gender in the analysis	0
Number of Studies considering social support as a variable in addition to gender in the analysis	0
Number of Studies considering migration as a variable in addition to gender in the analysis	0
Number of Studies considering sexual orientation as a variable in addition to gender in the analysis	0
4. Power Dynamics, Agency and Resistance:	
Number of studies exploring how individuals acknowledge and accept their intersecting identities	0
Number of studies exploring how individuals face suppression or marginalization due to their intersecting identities	0
Number of studies exploring how individuals and communities emancipate themselves from intersecting vulnerabilities	0
Number of studies exploring how do individuals negotiate their multiple identities	0
Number of studies exploring resistance and advocacy against discriminatory practices	0
Number of studies exploring psychological and emotional responses of individuals experiencing intersectional vulnerabilities	0
Number of studies exploring how do intersecting identities influence individuals’ behavioural and lifestyle adaptations	0
5. Perception of Vulnerability:	
Number of studies exploring perceptions of vulnerability among individuals with intersecting identities	0
Number of studies exploring how do cultural norms and contextual factors shape perceptions of vulnerability	0
Number of studies exploring how perceptions of vulnerability among individuals with intersecting identities has evolved over time	0
Number of studies exploring how do individuals with intersecting identities perceive their own adaptive capacities	0
Number of studies exploring the perceptions of resilience and coping strategies among individuals with intersecting identities	0
6. Gendered Outcomes of Adaptive Responses to Vulnerabilities:	
Number of studies exploring how do adaptive responses differ across various genders?	0
Number of studies exploring the gender-specific barriers and facilitators in accessing resources and services for adaptation	0
Number of studies exploring how adaptive measures influence gender-specific health and safety outcomes	0
Number of studies exploring the economic and livelihood outcomes for various genders following adaptation interventions	0
Number of studies exploring how adaptive responses affect gender roles, social dynamics, and cultural practices within communities	0
Number of studies exploring how adaptive strategies promote gender equality and empower marginalized gender identities	0

All the studies gathered information on the participants’ biological sex but labelled it as gender. They used terms like male and female, as well as men and women, to represent gender in their findings. Although they provided sex-disaggregated results, these were inadequeately labelled as gender with no complimentary gender analysis.The studies reported differences in AMR’s impact on men and women but did not look at the intersection of gender and other social factors. Gender was often used interchangeably with sex and did not consider its interaction with social factors such as age, socioeconomic status, and ethnicity.

Regarding AMR, all included studies focused solely on TB, leaving other diseases unexplored. Furthermore, the analysis typically stopped at the association between biological sex and various health outcomes. The evidence base (as shown in Table 2) lacks exploration into how gender identity, the social construct of gender or societal norms create or perpetuate inequities in healthcare access, influence perceptions of risk, or dictate the adaptive mechanisms and coping strategies used to manage AMR. This gap is particularly evident in low-income settings, where women were found to have limited access to formal healthcare and were more likely to resort to informal sources for antibiotics, yet the compounded effects of these intersectional vulnerabilities remain unstudied. (Table 2).

Despite the intersectional gaps noted previously, the 14 included studies provided sufficient sex-disaggregated data to allow for a narrative synthesis of outcomes. As summarized in Table 3, while all included studies provided data based on biological sex, there was a notable absence of advanced statistical analysis to explore intersections with other social variables like age, ethnicity, or socioeconomic status. Furthermore, the current research landscape lacks mixed-methods approaches that could integrate these quantitative outcomes with qualitative insights into gendered experiences.

**Table 3:** Exploration of Individual Studies Reporting based on the Intersectionality Guide in the context of AMR

Disease	Author and year	Outcome	Risk of Bias	Intersectionality
Tuberculosis	Kumar et al.2024 [23]	Treatment interruption pattern	**Moderate**	**Collected information on biological sex and mislabelled it as gender with two categories (i.e.**,** male and female).** **Presented sex- disaggregated results. Intersection of various social and other factors was not explored.**
	Shewade et al.2017 [28]	Treatment attrition rates	**Moderate**	
	Shewade et al.2018 [29]	Delay in diagnostic testing	**Low**	
	Raizada et al. 2015 [32]	TB diagnosis accuracy and rifampicin resistance detection	** Low**	
	Raizada et al. 2018 [33]	TB diagnosis rates, rifampicin resistance level	**Low**	
	Sidharta et al.2018 [34]	Treatment access, Health care behaviour	**Moderate**	
	Htun et al.2018 [25]	Treatment delay, disease severity, treatment adherence, treatment outcomes.	**Low**	
	Oga-Omenka et al.2019 [21]	Treatment initiation rates and timeliness of treatment	**Low**	
	Timire et al.2019 [22]	Access to diagnosis testing.	**Moderate**	
	Hapolo et al.2019 [24]	Treatment delay and initiation, Risk factor for delayed treatment	**Moderate**	
	Izudi et al.2020 [26]	Treatment success rate, MDR/RR TB surveillance rates	**Moderate**	
	Jouego et al.2022 [27]	Treatment outcome	**Moderate**	
	P Arif et al.2021 [30]	Treatment outcome, survival rate of MDR-TB patients	**Moderate**	
	Kyrbashov et al.2023 [31]	Diagnosis and treatment initiation, Treatment outcome.	**Moderate**	

#### Methodological quality of included studies

The quality appraisal revealed a robust evidence base, with five studies demonstrating a Low Risk of Bias and nine studies demonstrating a Moderate Risk of Bias, as presented in Table 3.

#### Narrative synthesis of findings from included studies

14 studies reported sex-disaggregated results for AMR-related service delivery. All 14 studies were conducted on TB.

####  Sex parity in diagnostic access

Fourteen studies reported sex-disaggregated results for AMR-related service delivery, all focusing on TB. When assessing diagnostic access, the evidence suggests a high degree of sex parity in testing services. For instance, Shewade et al. (2018) [29], Izudi et al. (2020) [26], and Timire et al. (2019) [22] found no significant difference in testing delays between sexes, with median durations from symptom onset to testing remaining remarkably similar (7 days for males vs. 8 days for females). Additionally, Raizada et al. (2015) [32] found no significant difference in the incidence of rifampicin-resistant TB when providing upfront molecular testing (2.4% for females vs. 2.0% for males).

#### Inconsistent Sex and Gender related risks in treatment initiation

The evidence regarding the risk of delay in treatment initiation was mixed across the included literature. Some contexts identified significant gendered vulnerabilities; Htun et al. (2018) [25] reported that females faced a substantially higher risk for longer treatment delays compared to males (65.5% vs. 43.9%). However, this finding was not universal. Both Ogaomenka et al. (2019) [21] and Hapolo et al. (2019) [24] stated no difference between the sexes. This parity was further supported by Raizada et al. (2018) [33], whose assessment of treatment initiation proportions found no significant difference (males 90.3% vs. females 89.4%), suggesting that gendered power dynamics did not obstruct the start of therapy in that specific study population.

#### Male vulnerability in retention and mortality outcomes

When analysing long-term outcomes, several studies identified a consistent trend of higher risk among male patients. While some metrics like treatment interruption showed little variance (Kumar et al. 2024 [23] reported 52% for males vs. 51% for females), males were more likely to experience pre-treatment attrition (40% vs. 29% in Shewade et al. 2017) [28]. Most significantly, three studies Kyrbashov et al. (2023) [33], Arif et al. (2021) [30], and Jouego et al. (2022) [27] collectively found that males were at a higher risk for unsuccessful outcomes, including a 4.06-fold higher risk of mortality. These outcomes may be linked to treatment-seeking behaviour, as Sidharta et al. (2018) [34] observed that males were less likely to report to private practitioners as a first point of contact.

## Discussion

The intersection between sex, gender identity, gender roles, and gender norms and AMR presents a critical challenge in LICs and LMICs, where existing health inequities are exacerbated by overlapping social factors. Although gender, as socially constructed determinant, influences health-seeking behaviour, exposure to risk factors, and access to healthcare services, many existing studies on AMR still conflate sex and gender, failing to recognize their distinct roles and the ways they interact with other social factors.

The most striking finding of this evidence gap map is the absolute thematic concentration on TB, contrasted by a total absence of intersectional AMR research concerning Malaria and NTDs in LLMICs. While the search strategy was exhaustive encompassing over 20 NTDs the zero-yield for these conditions highlights a “conceptual vacuum” in how AMR is currently studied outside of the TB program. This concentration suggests that while AMR is a global threat, research infrastructure in low-resource settings remains siloed, with intersectional gender analysis predominantly tethered to high-burden, vertically funded programs like TB.

Several factors may explain why Malaria and NTDs are missing from the current intersectional evidence base. Unlike TB programs with long-standing requirements for sex-disaggregated reporting, many NTD studies continue to report aggregate data, which precludes their inclusion in intersectional reviews. Research into Malaria and NTDs often prioritizes environmental and biological vectors, frequently overlooking the social and gendered power dynamics that dictate exposure risks and treatment-seeking behaviour. Current literature, as quantified in Table 2, largely conflates gender and sex, reducing gender to a binary biological variable (“sex”). This also fails to capture the complex identity markers such as age, occupation, and migration status that are essential for understanding AMR in the broader context of infectious diseases of poverty.

This review also revealed that while some studies provided sex-disaggregated data, they often did not examine how different contextual social locations, such as gender identity, age, socioeconomic status, and ethnicity, healthcare infrastructure, access, health seeking behaviour combine to influence AMR patterns. The intersections or interactions of these factors and how they compound one other can be studied in detail using statistical techniques (e.g., regression with interaction, cross classification, decision trees and multilevel analysis of individual heterogeneity [MAIHDA]) [35]. The lack of such analyses restrict our understanding of how the local context, and the social and gender dynamics influence AMR-related outcomes, particularly in diverse socio-economic and cultural settings.

The studies reviewed primarily focused on TB and showed a consistent gap in analysing how various social factors compound the inequities and lead to vulnerabilities. For instance, while the data from studies highlighted sex differences in healthcare access,^21–34^ these studies rarely explored how these differences might be further stratified by factors such as age, economic status, or geographical location. For example, women in low-income settings were found to have limited access to formal healthcare and were more likely to seek antibiotics from informal sources, potentially contributing to the spread of AMR [36–38]. This limitation highlights the need for future research to incorporate an intersectionality approach to understand the interaction of various contextual factors leading to inequities and vulnerabilities. Also, the studies did not explore the various adaptive mechanisms and coping strategies adopted by people to prevent or overcome the effects of AMR and how they can be influenced by gender and other social factors.

Furthermore, most studies presented sex-disaggregated data inadequately, labeling biological sex as gender without considering the implications of this misclassification. This conflation overlooks the broader social and cultural contexts in which gender operates, obscuring how gender-related power relations, roles, and norms impact health outcomes related to AMR [37, 39]. Addressing this misclassification is essential for ensuring that future research accurately reflects how gendered norms, roles and responses lead to other vulnerabilities that impact AMR. While the included studies provided sex-disaggregated data, they did not offer a critical analysis of how these results were shaped by intersecting social factors in context. In addition , it is important to recognize that gender does not operate in isolation. For example, a woman’s reliance on informal antibiotic markets can be seen as a result of the intersection between her role as a primary caregiver and her limited financial agency. When these factors are further compounded by geographic isolation, manageable diagnostic delays can transform into permanent treatment interruptions. By documenting that the current literature fails to examine these specific mechanisms, our study underscores the urgent need for future research to move beyond binary sex-disaggregated reporting and adopt a deeper intersectional lens.

Moreover, the review underscored a critical gap in research designs, with most studies adopting cross-sectional or retrospective approaches that provide only a static view of current conditions without capturing the evolving nature of the risk factors, their interactions and the inequities and vulnerabilities they create over time. This is particularly problematic in the AMR context, where factors influencing resistance patterns and healthcare access can change rapidly due to economic, social, and environmental conditions and shifts. Longitudinal studies are urgently needed to understand how gender and other social factors in context shape the health outcomes related to AMR. Additionally, the lack of mixed-methods research that integrates quantitative data with qualitative insights limits in-depth understanding of how social factors affect health behaviours and outcomes. Incorporating qualitative methods could provide richer contextual information to complement the quantitative data, leading to comprehensive understanding and analysis of gendered health disparities.

Stakeholder consultations conducted as part of this study highlighted the urgent need to incorporate an intersectionality approaches into both research and policy development to address AMR effectively. Experts also emphasized the importance of understanding how gender intersects with other social factors to shape health outcomes and inform intervention strategies. They called for tailored public health initiatives that consider these factors, particularly in designing gender transformative and equitable healthcare services [40] and community-based health monitoring systems, which is in line with the existing literature [41, 42]. This will help us understand how the experience of marginalised or disadvantaged populations is shaped by gender and other social factors in the context of AMR [43]. 

To address these gaps, future research should prioritize the application and adaptation of intersectional frameworks that capture the complex overlapping and simultaneous interactions between gender, social factors, systems and structures of power and AMR in different contexts and realities. Researchers should employ both quantitative and qualitative methods to explore these intersections and provide a comprehensive understanding of health disparities influenced by multiple levels of social injustice. Additionally, policy interventions should be informed by evidence generated from such research, ensuring they are inclusive and responsive to the diverse needs of different social groups and subgroups.

This review showcases several strengths, particularly its comprehensive and structured approach following PRISMA 2020 guidelines. The development of “Guide for an Intersectionality Approach to AMR, Gender, and Health Inequities” to explore the compounded impact of gender and social factors on AMR in LMICs and LICs is an additional strength of this study. Despite its robust methodology, the review has some limitations. While our search strategy was designed to encompass the full range of infectious diseases of poverty, all included studies focused solely on TB. This suggests a significant evidence gap in intersectional gender research for other critical disease areas affected by AMR, such as Malaria and NTDs. Consequently, this limits the generalizability of our findings across the broader spectrum of infectious diseases of poverty. While no formal language exclusion criteria were applied during the initial search setup, the databases utilised for this review are predominantly English-language repositories. Consequently, the resulting evidence base consisted only of studies published in the English language. Given the heterogeneity between included studies and broader nature of research questions, it was not possible to do meta-analysis.

## Conclusion

This review underscores the urgent need to incorporate an intersectional lens in addressing AMR in LICs and LMICs, as sex, gender identity, gender roles, and norms significantly influence health-seeking behaviours, healthcare access, and AMR-related risks. However, most studies conflate sex and gender, overlook intersecting social factors, and predominantly focus on TB, limiting a broader understanding of AMR across diverse diseases. The absence of advanced statistical techniques, longitudinal research, and mixed-methods approaches further constrains insights into how compounded vulnerabilities shape AMR disparities. Future research must integrate intersectional frameworks and mixed methodologies to capture these complexities, while policies should adopt gender-transformative approaches to ensure equitable healthcare access and effective antimicrobial stewardship. By addressing these gaps, AMR mitigation efforts can be more inclusive, context-specific, and sustainable in reducing health inequities.

## Supplementary Information

Supplementary Material 1.

## Data Availability

All the relevant data are presented within the manuscript. No additional data generated.

## Abbreviations

AMR: Antimicrobial Resistance
HIV: Human Immunodeficiency Virus
LIC: Low income countries
LMIC: Low middle income countries
NTD: Neglected Tropical Diseases
PRISMA: Preferred Reporting Items for Systematic Review and Meta-Analysis
TB: Tuberculosis

## Declarations

### Use of artificial intelligence tools

None declared.
