# Supplementary material for: Gender and other intersecting factors in antimicrobial resistance for infectious diseases of poverty: a systematic evidence gap analysis in low- and lower-middle-income countries
Source: BMC Public Health. 2026 Mar 16;26:1322. doi: 10.1186/s12889-026-26990-5 (PMC13104491; doi:10.1186/s12889-026-26990-5)
Supplement: Supplementary file 1 — Supplementary Material 1. [file 12889_2026_26990_MOESM1_ESM.pdf]

Supplementary File: Search Strategy

EMBASE:

Sl.no	Search strategy	Number of articles
1.	'gender and sex'/exp OR 'gender and sex' OR 'female'/exp OR 'female' OR 'females' OR 'woman' OR 'women' OR men OR 'gender identity'/exp OR 'gender identity' OR 'gender self-identification' OR 'identity, sexual' OR 'sex identification' OR 'sexual identification' OR 'sexual identity' OR 'sexual self-identification' OR 'identity'/exp OR 'crisis, identity' OR 'identity' OR 'identity crisis' OR 'sex'/exp OR 'sex' OR 'disability'/exp OR 'sexuality'/exp OR 'psychosexuality' OR 'sexual functioning' OR 'sexual habit' OR 'sexual hygiene' OR 'sexual partner' OR 'sexual partners' OR 'sexual reinforcement' OR 'sexual relation' OR 'sexuality' OR 'disability discrimination'/exp OR 'able-ism' OR 'ableism' OR 'disability discrimination' OR 'disability prejudice' OR 'disability-based discrimination' OR 'disability-related discrimination' OR 'disableism' OR 'handicap discrimination' OR 'racism'/exp OR 'discrimination, race' OR 'ethnic bias' OR 'ethnic discrimination' OR 'ethnic prejudice' OR 'ethnicism' OR 'ethnicity-based discrimination' OR 'ethnicity-related discrimination' OR 'race discrimination' OR 'race-based discrimination' OR 'race-related discrimination' OR 'racial bias' OR 'racial biases' OR 'racial discrimination' OR 'racial prejudice' OR 'racially motivated discrimination' OR 'racially-based discrimination' OR 'racism' OR 'racist behavior' OR 'racist behaviour' OR 'racist bias' OR 'racist prejudice' OR 'politics'/exp OR 'medical	16780519

	political action committee' OR 'politic' OR 'political factor' OR 'politics' OR 'capitalism'/exp OR 'capitalism' OR 'discrimination'/exp OR 'social status'/exp OR 'social achievement' OR 'social condition' OR 'social conditions' OR 'social economic status' OR 'social employment' OR 'social function' OR 'social importance' OR 'social rank' OR 'social standing' OR 'social state' OR 'social status' OR 'socio-economic status' OR 'socioeconomic status' OR 'status, social' OR 'residence characteristics'/exp OR 'place of residence' OR 'residence characteristics' OR 'residential address' OR 'residential characteristics' OR 'residential place' OR caste OR 'social class'/exp OR 'class, social' OR 'social class' OR 'sociocultural class' OR 'socioeconomic class' OR 'class background' OR 'refugee'/exp OR 'religion'/exp OR 'homophobia'/exp OR 'discrimination against gays' OR 'discrimination against homosexuals' OR 'discrimination against lesbians' OR 'gayphobia' OR 'homo-phobia' OR 'homophobia' OR 'homophobic attitude' OR 'homophobic behavior' OR 'homophobic behaviour' OR 'homophobic bias' OR 'homophobic discrimination' OR 'homophobic fears' OR 'homophobic prejudice' OR 'homophobic reaction' OR 'lesbophobia' OR 'lesbophobic attitude' OR 'ageism'/exp OR 'age discrimination' OR 'age prejudice' OR 'age-based discrimination' OR 'age-based prejudice' OR 'age-ism' OR 'age-related discrimination' OR 'age-related prejudice' OR 'ageism' OR 'discrimination based on age' OR 'transphobia'/exp OR wae OR 'war'/exp OR 'afghan campaign 2001-' OR 'american civil war' OR 'american revolution' OR 'crimean war' OR 'french revolution' OR 'gulf war' OR 'iraq war, 2003 -' OR 'iraq	

	war, 2003-2011' OR 'korean war' OR 'russian-japanese war' OR 'second world war' OR 'sino-japanese war' OR 'spanish-american war, 1898' OR 'vietnam conflict' OR 'world war i' OR 'world war ii' OR 'armed conflict' OR 'armed conflicts' OR 'war' OR 'war-time' OR 'wartime' OR 'sexism'/exp OR 'immigration'/exp OR 'education'/exp OR ex OR 'economic aspect'/exp OR classism	
2.	('neglected tropical disease'/exp OR 'buruli ulcer'/exp OR 'bairnsdale ulcer' OR 'buruli disease' OR 'buruli ulcer' OR 'buruli`s ulcer' OR 'daintree ulcer' OR 'mycobacterium ulcerans infection' OR 'mycobacterium ulcerans ulcer' OR 'searls ulcer' OR 'infection by m. ulcerans' OR 'infection by mycobacterium ulcerans' OR 'infection due to m. ulcerans' OR 'infection due to mycobacterium ulcerans' OR 'skin ulcer due to mycobacterium ulcerans' OR 'ulcer due to mycobacterium ulcerans' OR 'ulcer, buruli' OR 'chagas disease'/exp OR 'dengue'/exp OR 'aden fever' OR 'denv infection' OR 'denv virus infection' OR 'bouquet fever' OR 'break-bone fever' OR 'breakbone fever' OR 'classical dengue' OR 'classical dengue fever' OR 'dandy fever' OR 'dengue' OR 'dengue fever' OR 'dengue virus infection' OR 'infection by denv' OR 'infection by dengue virus' OR 'infection caused by dengue virus' OR 'solar fever' OR 'sun fever' OR 'chikungunya'/exp OR 'chikv infection' OR 'chickungunya' OR 'chikungunya' OR 'chikungunya disease' OR 'chikungunya fever' OR 'chikungunya virus (chikv) infection' OR 'chikungunya virus infection' OR 'infection by chikv' OR 'infection by chikungunya virus' OR 'infection caused by chikv' OR 'infection caused by chikungunya virus' OR	1191728

	'dracunculiasis'/exp OR 'dracunculus infection' OR 'dracunculus medinensis infection' OR 'guinea worm disease' OR 'guinea worm infection' OR 'dracontiasis' OR 'dracunculiasis' OR 'dracunculosis' OR 'guineaworm disease' OR 'guineaworm infection' OR 'echinococcosis'/exp OR 'echinococcus cyst' OR 'echinococcus disease' OR 'echinococcus granulosus cyst' OR 'echinococcus granulosus infection' OR 'echinococcus granulosus infestation' OR 'echinococcus infection' OR 'echinococcus infections' OR 'echinococcus infestation' OR 'cystic echinococcosis' OR 'echinococcal cyst' OR 'echinococcal cysts' OR 'echinococcal disease' OR 'echinococcal infection' OR 'echinococcal infections' OR 'echinococcal infestation' OR 'echinococciasis' OR 'echinococcoses' OR 'echinococcosis' OR 'hydatic cyst' OR 'hydatic echinococcosis' OR 'hydatid cyst' OR 'hydatid cystic disease' OR 'hydatid disease' OR 'hydatid disorder' OR 'hydatid echinococcus' OR 'hydatidiasis' OR 'hydatidosis' OR 'infection by echinococcus granulosus' OR 'infection of echinococcus granulosus' OR 'foodborne trematodes' OR 'trypanosomiasis'/exp OR 'trypanosoma infection' OR 'trypanosoma infections' OR 'infection by trypanosomes' OR 'trypanosomal infection' OR 'trypanosomal infections' OR 'trypanosome infection' OR 'trypanosome infections' OR 'trypanosomiases' OR 'trypanosomiasis' OR 'trypanosomoses' OR 'trypanosomosis' OR 'african trypanosomiasis'/exp OR 'african human trypanosomiasis' OR 'african lethargy' OR 'african sleeping sickness' OR 'african trypanosomiasis' OR 'congo trypanosomiasis' OR 'east african trypanosomiasis' OR 'gambian sleeping sickness' OR 'gambian	

	trypanosomiasis' OR 'gambian trypanosomosis' OR 'negro lethargy' OR 'rhodesian sleeping sickness' OR 'rhodesian trypanosomiasis' OR 'trypanosoma brucei gambiense infection' OR 'trypanosoma brucei infection' OR 'trypanosoma brucei rhodesiense infection' OR 'trypanosoma gambiense infection' OR 'trypanosoma rhodesiense infection' OR 'west african trypanosomiasis' OR 'human african trypanosomiasis' OR 'human african trypanosomosis' OR 'infection by trypanosoma brucei' OR 'infection of trypanosoma brucei' OR 'nelavan' OR 'sleeping sickness' OR 'trypanosomiasis, african' OR 'leishmaniasis'/exp OR 'leishmania infection' OR 'leishmania infections' OR 'infection by leishmania' OR 'infection of leishmania' OR 'leishmanial infection' OR 'leishmanial infections' OR 'leishmaniases' OR 'leishmaniasis' OR 'leishmaniosis' OR 'leprosy'/exp OR 'hansen disease' OR 'hansen`s disease' OR 'hansens disease' OR 'm. leprae infection' OR 'mycobacterium leprae infection' OR 'elephantiasis graecorum' OR 'hanseniasis' OR 'infection by m. leprae' OR 'infection by mycobacterium leprae' OR 'infection caused by m. leprae' OR 'infection caused by mycobacterium leprae' OR 'infection of mycobacterium leprae' OR 'lepra' OR 'leprology' OR 'leprosies' OR 'leprosis' OR 'leprosy' OR 'leprous infection' OR 'morbus hansen' OR 'elephantiasis'/exp OR 'congenital elephantiasis' OR 'elephanthiasis' OR 'elephantiases' OR 'elephantiasis' OR 'elephantiasis tropica' OR 'elephantiasis vulvae' OR 'mal de cayenne' OR 'sarcoma mucosum' OR 'spargosis fibro areolis' OR 'lymph flow'/exp OR 'circulation, lymph' OR 'flow, lymph' OR 'lymph circulation' OR 'lymph flow' OR	

	'lymphatic circulation' OR 'lymphatic filling' OR 'lymphatic flow' OR 'lymphatic spread' OR 'lymphokinesis' OR 'mycetoma'/exp OR 'mycetoma' OR 'mycetoma pedis' OR 'chromomycosis'/exp OR 'fonsecaea pedrosoi infection' OR 'phialophora verrucosa infection' OR 'chromoblastomycoses' OR 'chromoblastomycosis' OR 'chromomycoses' OR 'chromomycosis' OR 'dermatitis, verrucosa' OR 'verrucous dermatitis' OR 'mycosis'/exp OR 'deep mycosis' OR 'disease caused by fungi' OR 'disease caused by fungus' OR 'fungal disease' OR 'fungal diseases' OR 'fungal infection' OR 'fungal infections' OR 'fungal infectious disease' OR 'fungal infectious diseases' OR 'fungus disease' OR 'fungus diseases' OR 'fungus infection' OR 'infection caused by fungi' OR 'infection caused by fungus' OR 'mycoses' OR 'mycosis' OR 'mycosis infection' OR 'mycotic disease' OR 'mycotic diseases' OR 'mycotic infection' OR 'mycotic infections' OR 'onchocerciasis'/exp OR 'o. volvulus infection' OR 'onchocerca infection' OR 'onchocerca infections' OR 'onchocerca volvulus infection' OR 'infection by onchocerca volvulus' OR 'oncho-cerciasis' OR 'onchocerciasis' OR 'onchocercoses' OR 'onchocercosis' OR 'onchocerosis' OR 'volvulosis' OR noma OR 'rabies'/exp OR 'hubert disease' OR 'lyssavirus infection' OR 'bart rabies' OR 'control, rabies' OR 'disease, hubert' OR 'human rabies' OR 'infection by rabies virus' OR 'infection caused by rabies virus' OR 'lyssa' OR 'lyssa humana' OR 'lyssaviral infection' OR 'rabbia' OR 'rabies' OR 'rabies control' OR 'rabies diagnosis' OR 'rabies infection' OR 'rabies viral infection' OR 'rabies virus infection' OR 'scabies'/exp OR 's. scabiei infection' OR 's.	

	scabiei infestation' OR 'sarcoptes scabiei infection' OR 'sarcoptes scabiei infestation' OR 'infestation by sarcoptes scabiei' OR 'scabies' OR 'scabies, watchmaker' OR 'watchmaker scabies' OR 'ectoparasitosis'/exp OR 'ectoparasite infection' OR 'ectoparasite infections' OR 'ectoparasite infestation' OR 'ectoparasite infestations' OR 'ectoparasitic disease' OR 'ectoparasitic diseases' OR 'ectoparasitic infection' OR 'ectoparasitic infections' OR 'ectoparasitic infestation' OR 'ectoparasitic infestations' OR 'ectoparasitoses' OR 'ectoparasitosis' OR 'schistosomiasis'/exp OR 'schistosoma infection' OR 'schistosoma infections' OR 'bilharzia infection' OR 'bilharziasis' OR 'bilharzioses' OR 'bilharziosis' OR 'blood fluke infection' OR 'infection by schistosoma' OR 'schistomiasis' OR 'schistosomatosis' OR 'schistosome infection' OR 'schistosomiases' OR 'schistosomiasis' OR 'schistosomosis' OR 'snail fever' OR 'helminthiasis'/exp OR 'helminth disease' OR 'helminth diseases' OR 'helminth infection' OR 'helminth infections' OR 'helminth infestation' OR 'helminth infestations' OR 'helminth parasitic infection' OR 'helminth parasitic infections' OR 'helminthiases' OR 'helminthiasis' OR 'helminthic disease' OR 'helminthic infection' OR 'helminthic infections' OR 'helminthic infestation' OR 'helminthic infestations' OR 'helminthoses' OR 'helminthosis' OR 'infection with helminths' OR 'infection with parasitic worms' OR 'infection, helminthic' OR 'infection, worm' OR 'infections with helminths' OR 'parasitic worm infection' OR 'parasitic worm infestation' OR 'worm disease' OR 'worm diseases' OR 'worm infection' OR 'worm infections' OR 'worm infestation' OR 'worm	

	infestations' OR 'snakebite'/exp OR 'snake bite' OR 'snake bites' OR 'snakebite' OR taeniasis OR 'cysticercosis'/exp OR 'cysticerciasis' OR 'cysticercoses' OR 'cysticercosis' OR 'larval taeniasis' OR 'larval tapeworm infection' OR 'larval tapeworm infections' OR 'trachoma'/exp OR 'egyptian ophthalmia' OR 'conjunctivitis, granular' OR 'granular conjunctivitis' OR 'trachoma' OR 'trachomatous trichiasis' OR 'yaws'/exp OR 't. pertenue infection' OR 'treponema pertenue infection' OR 'frambesia' OR 'framboesia' OR 'framboesia tropica' OR 'parangi' OR 'pian' OR 'yaw' OR 'yaws' OR 'tuberculosis'/exp OR 'koch`s disease' OR 'm. tuberculosis infection' OR 'mycobacterium tuberculosis infection' OR 'tb (tuberculosis)' OR 'tb case' OR 'tb cases' OR 'tb disease' OR 'tb infection' OR 'active tb' OR 'active tuberculosis' OR 'case of tb' OR 'cases of tb' OR 'chronic tuberculosis' OR 'infection by m. tuberculosis' OR 'infection by mycobacterium tuberculosis' OR 'infection due to m. tuberculosis' OR 'infection due to mycobacterium tuberculosis' OR 'infection of m. tuberculosis' OR 'infection of mycobacterium tuberculosis' OR 'minimal tuberculosis' OR 'minimum tuberculosis' OR 'tuberculosis' OR 'tuberculous infection' OR 'tuberculous lesion' OR 'malaria'/exp OR 'plasmodia infection' OR 'plasmodium infection' OR 'gametocytaemia' OR 'gametocytemia' OR 'infection by plasmodium' OR 'malaria' OR 'malaria infection' OR 'malaria transmission' OR 'malarial fever' OR 'malarial infection' OR 'marsh fever' OR 'paludism' OR 'plasmodial infection' OR 'plasmodiosis' OR 'swamp fever (malaria)')	

3.	'antineoplastic agent resistance' OR 'antineoplastic drug resistance' OR 'chemical resistance' OR 'chemoresistance' OR 'chemotherapy, drug resistance' OR 'drug resistance' OR 'drug resistance, neoplasm' OR 'resistance, drug' OR 'drug resistance'/exp OR 'antibiotic resistance'/exp OR 'antibacterial drug resistance' OR 'antibacterial resistance' OR 'antibiotic non-susceptibility' OR 'antibiotic nonsusceptibility' OR 'antibiotic resistance' OR 'antimicrobial drug resistance' OR 'antimicrobial resistance' OR 'bacterial drug resistance' OR 'bacterial resistance' OR 'bacterium resistance' OR 'drug resistance, bacterial' OR 'drug resistance, microbial' OR 'microbial drug resistance' OR 'resistance, antibiotic'	586523
4.	#1 AND #2 AND #3	26246

Scopus:

(("Drug Resistance, Microbial" OR "Drug Resistances, Microbial" OR "Antimicrobial Drug Resistance" OR "Antimicrobial Drug Resistances" OR "Antimicrobial Resistance, Drug" OR "Antimicrobial Resistances, Drug" OR "Drug Antimicrobial Resistance" OR "Drug Antimicrobial Resistances" OR "Resistance, Drug Antimicrobial" OR "Resistances, Drug Antimicrobial" OR "Antibiotic Resistance, Microbial" OR "Antibiotic Resistance" OR "Resistance, Antibiotic") AND ("gender identity" OR ("gender identity" OR ("gender" AND "identity") OR "gender identity" OR "gender s" OR "genders" OR "sex" OR "sex" OR "gender") OR "sex" OR elder* OR ability OR "Disability Discrimination" OR disability OR ethnicity OR sexuality OR education OR ableism OR race OR racism OR politics OR capitalism OR discrimination OR socioeconomic status OR "Residence Characteristics" OR marginalized OR caste OR social class OR "class background" OR refugee OR religion OR

homophobia OR ageism OR transphobia OR ethnocentrism OR heterosexism OR sexism OR war OR immigration OR education OR economy OR classism) AND ("Tuberculosis" OR "Extensively Drug-Resistant Tuberculosis" OR "Tuberculosis, Multidrug-Resistant" OR "TB" OR "Tuberculosis" OR "Extensively Drug-Resistant Tuberculosis" OR " Multidrug-Resistant Tuberculosis" OR "Neglected Diseases" OR "Buruli ulcer" OR "Chagas disease" OR dengue OR chikungunya OR dracunculiasis OR echinococcosis OR "foodborne trematodiases" OR "human African trypanosomiasis" OR leishmaniasis OR leprosy OR "lymphatic filariasis" OR mycetoma OR chromoblastomycosis OR "deep mycoses" OR noma OR onchocerciasis OR rabies OR scabies OR ectoparasitoses OR schistosomiasis OR "soil-transmitted helminthiases" OR "snakebite envenoming" OR taeniasis OR cysticercosis OR trachoma OR yaws OR "Malaria" OR "Marsh Fever" OR "Fever, Marsh" OR Paludism OR "Remittent Fever" OR "Fever, Remittent" OR "Infections, Plasmodium" OR "Infection, Plasmodium" OR "Plasmodium Infection" OR "Plasmodium Infections" OR "Malaria")) AND (LIMIT-TO (DOCTYPE,"ar") OR LIMIT-TO (DOCTYPE,"re")) AND (LIMIT-TO (SRCTYPE,"j")) – 3218 results

PubMed:

Query No.	Search Terms	Results
#1	"drug resistance, microbial"[MeSH Terms] OR ("drug resistance, microbial"[MeSH Terms] OR ("drug"[All Fields] AND "resistance"[All Fields] AND "microbial"[All Fields]) OR "microbial drug resistance"[All Fields] OR ("drug"[All Fields] AND "resistances"[All Fields] AND "microbial"[All Fields])) OR "Antimicrobial Drug Resistance"[All Fields] OR "Antimicrobial Drug Resistances"[All Fields] OR ("drug resistance, microbial"[MeSH Terms] OR ("drug"[All Fields] AND "resistance"[All Fields] AND "microbial"[All Fields]) OR "microbial drug resistance"[All Fields] OR ("antimicrobial"[All Fields] AND "resistance"[All Fields] AND "drug"[All Fields]) OR "antimicrobial resistance drug"[All Fields]) OR ("drug resistance, microbial"[MeSH Terms] OR ("drug"[All Fields] AND "resistance"[All Fields] AND "microbial"[All Fields]) OR "microbial drug resistance"[All Fields] OR ("antimicrobial"[All Fields] AND "resistances"[All Fields] AND "drug"[All Fields])) OR ("drug resistance, microbial"[MeSH Terms] OR ("drug"[All Fields] AND "resistance"[All Fields] AND "microbial"[All Fields]) OR "microbial drug resistance"[All Fields] OR ("drug"[All Fields] AND "antimicrobial"[All Fields] AND "resistance"[All Fields]) OR "drug antimicrobial resistance"[All Fields]) OR ("drug resistance, microbial"[MeSH Terms] OR ("drug"[All Fields] AND "resistance"[All Fields] AND "microbial"[All Fields]) OR "microbial	247,544

	drug resistance"[All Fields] OR ("drug"[All Fields] AND "antimicrobial"[All Fields] AND "resistances"[All Fields])) OR ("drug resistance, microbial"[MeSH Terms] OR ("drug"[All Fields] AND "resistance"[All Fields] AND "microbial"[All Fields]) OR "microbial drug resistance"[All Fields] OR ("resistance"[All Fields] AND "drug"[All Fields] AND "antimicrobial"[All Fields])) OR ("drug resistance, microbial"[MeSH Terms] OR ("drug"[All Fields] AND "resistance"[All Fields] AND "microbial"[All Fields]) OR "microbial drug resistance"[All Fields] OR ("resistances"[All Fields] AND "drug"[All Fields] AND "antimicrobial"[All Fields])) OR "antibiotic resistance microbial"[All Fields] OR "Antibiotic Resistance"[All Fields] OR "resistance antibiotic"[All Fields]	
#2	"gender identity"[MeSH Terms] OR (((("gender identity"[MeSH Terms] OR ("gender"[All Fields] AND "identity"[All Fields]) OR "gender identity"[All Fields]) AND "O"[All Fields]) AND "gender s"[All Fields]) OR "genders"[All Fields] OR "sex"[MeSH Terms] OR "sex"[All Fields] OR "gender"[All Fields]) OR "sex"[MeSH Terms] OR ("sex"[MeSH Terms] OR "sex"[All Fields]) OR "elder*"[MeSH Terms] OR "aptitude"[MeSH Terms] OR "Disability Discrimination"[MeSH Terms] OR "disability"[Title/Abstract] OR ("ethnology"[MeSH Terms] OR "ethnicity"[MeSH Terms]) OR ("sexuality"[MeSH Terms] OR "sexual behavior"[MeSH Terms]) OR ("educational status"[MeSH Terms] OR "education"[MeSH Terms]) OR "Disability Discrimination"[MeSH Terms] OR "racial groups"[MeSH Terms] OR "racism"[MeSH Terms] OR	33,88,899

	"politics"[MeSH Terms] OR "capitalism"[MeSH Terms] OR "discrimination, psychological"[MeSH Terms] OR "social class"[MeSH Terms] OR "Residence Characteristics"[MeSH Terms] OR "marginalized"[Title/Abstract] OR "social class"[MeSH Terms] OR "social class"[MeSH Terms] OR "class background"[Title/Abstract] OR "refugees"[MeSH Terms] OR "religion"[MeSH Terms] OR "homophobia"[MeSH Terms] OR "ageism"[MeSH Terms] OR "transphobia"[All Fields] OR ("ethnocentric"[All Fields] OR "ethnocentricity"[All Fields] OR "ethnocentrism"[All Fields]) OR "heterosexism"[All Fields] OR "sexism"[MeSH Terms] OR "armed conflicts"[MeSH Terms] OR "emigration and immigration"[MeSH Terms] OR ("educational status"[MeSH Terms] OR "education"[MeSH Terms]) OR "economics"[MeSH Terms] OR "classism"[All Fields]	
#3	"Tuberculosis"[MeSH Terms] OR "Extensively Drug-Resistant Tuberculosis"[MeSH Terms] OR "tuberculosis, multidrug resistant"[MeSH Terms] OR "TB"[Title/Abstract] OR "Tuberculosis"[Title/Abstract] OR "Extensively Drug-Resistant Tuberculosis"[Title/Abstract] OR "Multidrug-Resistant Tuberculosis"[Title/Abstract] OR ("Neglected Diseases"[MeSH Terms] OR "buruli ulcer"[MeSH Terms] OR "buruli ulcer"[Title/Abstract] OR "chagas disease"[MeSH Terms] OR "chagas disease"[Title/Abstract] OR "dengue"[MeSH Terms] OR "dengue"[Title/Abstract] OR "chikungunya fever"[MeSH Terms] OR "chikungunya"[Title/Abstract] OR "dracunculiasis"[MeSH Terms]	622,905

	OR "dracunculiasis"[Title/Abstract] OR "echinococcosis"[MeSH Terms] OR "echinococcosis"[Title/Abstract] OR "foodborne trematodiases"[All Fields] OR (("human s"[All Fields] OR "humans"[MeSH Terms] OR "humans"[All Fields] OR "human"[All Fields]) AND "trypanosomiasis, african"[MeSH Terms]) OR "human african trypanosomiasis"[Title/Abstract] OR ("leishmaniasis"[MeSH Terms] OR "leishmaniasis vaccines"[MeSH Terms]) OR "leishmaniasis"[Title/Abstract] OR "leprosy"[MeSH Terms] OR "leprosy"[Title/Abstract] OR "elephantiasis, filarial"[MeSH Terms] OR "lymphatic filariasis"[Title/Abstract] OR "mycetoma"[MeSH Terms] OR "mycetoma"[Title/Abstract] OR "chromoblastomycosis"[MeSH Terms] OR "chromoblastomycosis"[Title/Abstract] OR ("deep"[All Fields] AND "mycoses"[MeSH Terms]) OR "deep mycoses"[Title/Abstract] OR "noma"[MeSH Terms] OR "noma"[Title/Abstract] OR "onchocerciasis"[MeSH Terms] OR "onchocerciasis"[Title/Abstract] OR "rabies"[MeSH Terms] OR "rabies"[Title/Abstract] OR "scabies"[MeSH Terms] OR "scabies"[Title/Abstract] OR "ectoparasitoses"[All Fields] OR "ectoparasitoses"[Title/Abstract] OR "schistosomiasis"[MeSH Terms] OR "schistosomiasis"[Title/Abstract] OR ("soil-transmitted"[All Fields] AND "helminthiasis"[MeSH Terms]) OR "soil transmitted helminthiases"[Title/Abstract] OR "snake bites"[MeSH Terms] OR "snakebite envenoming"[Title/Abstract] OR "taeniasis"[MeSH Terms] OR "taeniasis"[Title/Abstract] OR "cysticercosis"[MeSH	

	Terms] OR "cysticercosis"[Title/Abstract] OR "trachoma"[MeSH Terms] OR "trachoma"[Title/Abstract] OR "yaws"[MeSH Terms] OR "yaws"[Title/Abstract]) OR ("Malaria"[MeSH Terms] OR "marsh fever"[Title/Abstract] OR (("Fever"[MeSH Terms] OR "Fever"[All Fields] OR "fevers"[All Fields]) AND "Marsh"[Title/Abstract]) OR "Paludism"[Title/Abstract] OR "remittent fever"[Title/Abstract] OR "fever remittent"[Title/Abstract] OR "infections plasmodium"[Title/Abstract] OR "infection plasmodium"[Title/Abstract] OR "plasmodium infection"[Title/Abstract] OR "plasmodium infections"[Title/Abstract] OR "Malaria"[Title/Abstract])	
#4	#1 AND #2 AND #3	771

Web of Science:

SEARCH 1:
**(((**AB=(Tuberculosis) OR TI=(Tuberculosis) OR KP=(Tuberculosis) OR AB=(Extensively Drug-Resistant Tuberculosis) OR TI=(Extensively Drug-Resistant Tuberculosis) OR KP=(Extensively Drug-Resistant Tuberculosis) OR AB=(Tuberculosis, Multidrug-Resistant) OR TI=(Tuberculosis, Multidrug-Resistant) OR KP=(Tuberculosis, Multidrug-Resistant) OR AB=(Multidrug-Resistant Tuberculosis) OR TI=(Multidrug-Resistant Tuberculosis) OR KP=(Multidrug-Resistant Tuberculosis)**) OR (**AB=(Malaria) OR TI=(Malaria) OR KP=(Malaria) OR AB=(Marsh Fever) OR TI=(Marsh Fever) OR KP=(Marsh Fever) OR AB=(Paludism) OR TI=(Paludism) OR KP=(Paludism) OR

AB=(Remittent Fever) OR TI=(Remittent Fever) OR KP=(Remittent Fever) OR ALL=(Plasmodium) OR AB=(Plasmodium) OR TI=(Plasmodium) OR KP=(Plasmodium)) **OR (**ALL=(Neglected Diseases) OR AB=(Neglected Diseases) OR TI=(Neglected Diseases) OR KP=(Neglected Diseases) OR

ALL=(Neglected Tropical Diseases) OR AB=(Neglected Tropical Diseases) OR TI=(Neglected Tropical Diseases) OR KP=(Neglected Tropical Diseases) OR

ALL=(Buruli ulcer) OR AB=(Buruli ulcer) OR TI=(Buruli ulcer) OR KP=(Buruli ulcer) OR

ALL=(Chagas disease) OR AB=(Chagas disease) OR TI=(Chagas disease) OR KP=(Chagas disease) OR

ALL=(dengue) OR AB=(dengue) OR TI=(dengue) OR KP=(dengue) OR

ALL=(chikungunya) OR AB=(chikungunya) OR TI=(chikungunya) OR KP=(chikungunya) OR

ALL=(dracunculiasis) OR AB=(dracunculiasis) OR TI=(dracunculiasis) OR KP=(dracunculiasis) OR

ALL=(echinococcosis) OR AB=(echinococcosis) OR TI=(echinococcosis) OR KP=(echinococcosis) OR

ALL=(trematodiasis) OR AB=(trematodiasis) OR TI=(trematodiasis) OR KP=(trematodiasis) OR

ALL=(human African trypanosomiasis) OR AB=(trypanosomiasis) OR TI=(trypanosomiasis) OR KP=(trypanosomiasis) OR

ALL=(leishmaniasis) OR AB=(leishmaniasis) OR TI=(leishmaniasis) OR KP=(leishmaniasis) OR

ALL=(leprosy) OR AB=(leprosy) OR TI=(leprosy) OR KP=(leprosy) OR

ALL=(lymphatic filariasis) OR AB=(lymphatic filariasis) OR TI=(lymphatic filariasis) OR KP=(lymphatic filariasis) OR

ALL=(mycetoma) OR AB=(mycetoma) OR TI=(mycetoma) OR KP=(mycetoma) OR
ALL=(chromoblastomycosis) OR AB=(chromoblastomycosis) OR TI=(chromoblastomycosis) OR KP=(chromoblastomycosis) OR
ALL=(deep mycoses) OR AB=(deep mycoses) OR TI=(deep mycoses) OR KP=(deep mycoses) OR
ALL=(noma) OR AB=(noma) OR TI=(noma) OR KP=(noma) OR
ALL=(onchocerciasis) OR AB=(onchocerciasis) OR TI=(onchocerciasis) OR KP=(onchocerciasis) OR
ALL=(rabies) OR AB=(rabies) OR TI=(rabies) OR KP=(rabies) OR
ALL=(scabies) OR AB=(scabies) OR TI=(scabies) OR KP=(scabies) OR
ALL=(ectoparasitoses) OR AB=(ectoparasitoses) OR TI=(ectoparasitoses) OR KP=(ectoparasitoses) OR
ALL=(schistosomiasis) OR AB=(schistosomiasis) OR TI=(schistosomiasis) OR KP=(schistosomiasis) OR
ALL=(soil-transmitted helminthiases) OR AB=(soil-transmitted helminthiases) OR TI=(soil-transmitted helminthiases) OR KP=(soil-transmitted helminthiases) OR
ALL=(snakebite) OR AB=(snakebite) OR TI=(snakebite) OR KP=(snakebite) OR
ALL=(envenoming) OR AB=(envenoming) OR TI=(envenoming) OR KP=(envenoming) OR
ALL=(taeniasis) OR AB=(taeniasis) OR TI=(taeniasis) OR KP=(taeniasis) OR
ALL=(cysticercosis) OR AB=(cysticercosis) OR TI=(cysticercosis) OR KP=(cysticercosis) OR
ALL=(trachoma) OR AB=(trachoma) OR TI=(trachoma) OR KP=(trachoma) OR
ALL=(yaws) OR AB=(yaws) OR TI=(yaws) OR KP=(yaws)**)) AND**

((ALL=(gender identity) OR AB=(gender identity) OR TI=(gender identity) OR KP=(gender identity) OR

ALL=(gender) OR AB=(gender) OR TI=(gender) OR KP=(gender) OR

AB=(identity) OR TI=(identity) OR KP=(identity) OR

AB=(genders) OR TI=(genders) OR KP=(genders) OR

AB=(sex) OR TI=(sex) OR KP=(sex)

OR AB=(elder) OR TI=(elder) OR KP=(elder)

OR AB=(ability) OR TI=(ability) OR KP=(ability) OR

AB=(Disability Discrimination) OR TI=(Disability Discrimination) OR KP=(Disability Discrimination) OR

AB=(disability) OR TI=(disability) OR KP=(disability) OR

AB=(ethnicity) OR TI=(ethnicity) OR KP=(ethnicity) OR

AB=(sexuality) OR TI=(sexuality) OR KP=(sexuality) OR

AB=(education) OR TI=(education) OR KP=(education) OR

AB=(ableism) OR TI=(ableism) OR KP=(ableism) OR

AB=(race) OR TI=(race) OR KP=(race) OR

AB=(racism) OR TI=(racism) OR KP=(racism) OR

AB=(politics) OR TI=(politics) OR KP=(politics) OR

AB=(capitalism) OR TI=(capitalism) OR KP=(capitalism) OR

AB=(discrimination) OR TI=(discrimination) OR KP=(discrimination) OR

AB=(socioeconomic status) OR TI=(socioeconomic status) OR KP=(socioeconomic status) OR

AB=(Residence Characteristics) OR TI=(Residence Characteristics) OR KP=(Residence Characteristics) OR AB=(marginalized) OR TI=(marginalized) OR KP=(marginalized) OR

AB=(caste) OR TI=(caste) OR KP=(caste) OR

AB=(social class) OR TI=(social class) OR KP=(social class) OR

AB=(class background) OR TI=(class background) OR KP=(class background) OR

AB=(refugee) OR TI=(refugee) OR KP=(refugee) OR

AB=(religion) OR TI=(religion) OR KP=(religion) OR

AB=(homophobia) OR TI=(homophobia) OR KP=(homophobia) OR

AB=(ageism) OR TI=(ageism) OR KP=(ageism) OR

AB=(transphobia) OR TI=(transphobia) OR KP=(transphobia) OR

AB=(ethnocentrism) OR TI=(ethnocentrism) OR KP=(ethnocentrism) OR

AB=(heterosexism) OR TI=(heterosexism) OR KP=(heterosexism) OR

AB=(sexism) OR TI=(sexism) OR KP=(sexism) OR

AB=(war) OR TI=(war) OR KP=(war) OR

AB=(immigration) OR TI=(immigration) OR KP=(immigration) OR

AB=(economy) OR TI=(economy) OR KP=(economy) OR

AB=(classism) OR TI=(classism) OR KP=(classism)**)) AND ((**AB=(Drug Resistance, Microbial) OR TI=(Drug Resistance, Microbial) OR KP=(Drug Resistance, Microbial) OR

AB=(Drug Resistances, Microbial) OR TI=(Drug Resistances, Microbial) OR KP=(Drug Resistances, Microbial) OR

AB=(Antimicrobial Drug Resistance) OR TI=(Antimicrobial Drug Resistance) OR KP=(Antimicrobial Drug Resistance) OR

AB=(Antimicrobial Drug Resistances) OR TI=(Antimicrobial Drug Resistances) OR KP=(Antimicrobial Drug Resistances) OR

AB=(Antimicrobial Resistance, Drug) OR TI=(Antimicrobial Resistance, Drug) OR KP=(Antimicrobial Resistance, Drug) OR

AB=(Antimicrobial Resistances, Drug) OR TI=(Antimicrobial Resistances, Drug) OR KP=(Antimicrobial Resistances, Drug) OR

AB=(Drug Antimicrobial Resistance) OR TI=(Drug Antimicrobial Resistance) OR KP=(Drug Antimicrobial Resistance) OR<br>AB=(Drug Antimicrobial Resistances) OR TI=(Drug Antimicrobial Resistances) OR KP=(Drug Antimicrobial Resistances) OR<br>AB=(Resistance, Drug Antimicrobial) OR TI=(Resistance, Drug Antimicrobial) OR KP=(Resistance, Drug Antimicrobial) OR<br>AB=(Resistances, Drug Antimicrobial) OR TI=(Resistances, Drug Antimicrobial) OR KP=(Resistances, Drug Antimicrobial) OR<br>AB=(Antibiotic Resistance, Microbial) OR TI=(Antibiotic Resistance, Microbial) OR KP=(Antibiotic Resistance, Microbial) OR<br>AB=(Antibiotic Resistance) OR TI=(Antibiotic Resistance) OR KP=(Antibiotic Resistance) OR<br>AB=(Resistance, Antibiotic) OR TI=(Resistance, Antibiotic) OR KP=(Resistance, Antibiotic) OR<br>ALL=(Antibiotic Resistance) OR ALL=(Resistance)**)))**
SEARCH 2:
**(((**AB=(Tuberculosis) OR TI=(Tuberculosis) OR KP=(Tuberculosis) OR<br>AB=(Extensively Drug-Resistant Tuberculosis) OR TI=(Extensively Drug-Resistant Tuberculosis) OR KP=(Extensively Drug-Resistant Tuberculosis) OR<br>AB=(Tuberculosis, Multidrug-Resistant) OR TI=(Tuberculosis, Multidrug-Resistant) OR KP=(Tuberculosis, Multidrug-Resistant) OR<br>AB=(Multidrug-Resistant Tuberculosis) OR TI=(Multidrug-Resistant Tuberculosis) OR KP=(Multidrug-Resistant Tuberculosis)**) OR (**AB=(Malaria) OR TI=(Malaria) OR KP=(Malaria) OR<br>AB=(Marsh Fever) OR TI=(Marsh Fever) OR KP=(Marsh Fever) OR

AB=(Paludism) OR TI=(Paludism) OR KP=(Paludism) OR

AB=(Remittent Fever) OR TI=(Remittent Fever) OR KP=(Remittent Fever) OR

AB=(Plasmodium) OR TI=(Plasmodium) OR KP=(Plasmodium)**) OR (**ALL=(Neglected Diseases) OR AB=(Neglected Diseases) OR TI=(Neglected Diseases) OR KP=(Neglected Diseases) OR

AB=(Neglected Tropical Diseases) OR TI=(Neglected Tropical Diseases) OR KP=(Neglected Tropical Diseases) OR

AB=(Buruli ulcer) OR TI=(Buruli ulcer) OR KP=(Buruli ulcer) OR

AB=(Chagas disease) OR TI=(Chagas disease) OR KP=(Chagas disease) OR

AB=(dengue) OR TI=(dengue) OR KP=(dengue) OR

AB=(chikungunya) OR TI=(chikungunya) OR KP=(chikungunya) OR

AB=(dracunculiasis) OR TI=(dracunculiasis) OR KP=(dracunculiasis) OR

AB=(echinococcosis) OR TI=(echinococcosis) OR KP=(echinococcosis) OR

AB=(trematodiasis) OR TI=(trematodiasis) OR KP=(trematodiasis) OR

AB=(trypanosomiasis) OR TI=(trypanosomiasis) OR KP=(trypanosomiasis) OR

AB=(leishmaniasis) OR TI=(leishmaniasis) OR KP=(leishmaniasis) OR

AB=(leprosy) OR TI=(leprosy) OR KP=(leprosy) OR

AB=(lymphatic filariasis) OR TI=(lymphatic filariasis) OR KP=(lymphatic filariasis) OR

AB=(mycetoma) OR TI=(mycetoma) OR KP=(mycetoma) OR

AB=(chromoblastomycosis) OR TI=(chromoblastomycosis) OR KP=(chromoblastomycosis) OR

AB=(deep mycoses) OR TI=(deep mycoses) OR KP=(deep mycoses) OR

AB=(noma) OR TI=(noma) OR KP=(noma) OR

AB=(onchocerciasis) OR TI=(onchocerciasis) OR KP=(onchocerciasis) OR

AB=(rabies) OR TI=(rabies) OR KP=(rabies) OR

AB=(scabies) OR TI=(scabies) OR KP=(scabies) OR

AB=(ectoparasitoses) OR TI=(ectoparasitoses) OR KP=(ectoparasitoses) OR

AB=(schistosomiasis) OR TI=(schistosomiasis) OR KP=(schistosomiasis) OR

AB=(soil-transmitted helminthiases) OR TI=(soil-transmitted helminthiases) OR KP=(soil-transmitted helminthiases) OR

AB=(snakebite) OR TI=(snakebite) OR KP=(snakebite) OR

AB=(envenoming) OR TI=(envenoming) OR KP=(envenoming) OR

AB=(taeniasis) OR TI=(taeniasis) OR KP=(taeniasis) OR

AB=(cysticercosis) OR TI=(cysticercosis) OR KP=(cysticercosis) OR

AB=(trachoma) OR TI=(trachoma) OR KP=(trachoma) OR

AB=(yaws) OR TI=(yaws) OR KP=(yaws)**)) AND**

((ALL=(gender identity) OR AB=(gender identity) OR TI=(gender identity) OR KP=(gender identity) OR

ALL=(gender) OR AB=(gender) OR TI=(gender) OR KP=(gender) OR

AB=(identity) OR TI=(identity) OR KP=(identity) OR

AB=(genders) OR TI=(genders) OR KP=(genders) OR

AB=(sex) OR TI=(sex) OR KP=(sex)

OR AB=(elder) OR TI=(elder) OR KP=(elder)

OR AB=(ability) OR TI=(ability) OR KP=(ability) OR

AB=(Disability Discrimination) OR TI=(Disability Discrimination) OR KP=(Disability Discrimination) OR

AB=(disability) OR TI=(disability) OR KP=(disability) OR

AB=(ethnicity) OR TI=(ethnicity) OR KP=(ethnicity) OR

AB=(sexuality) OR TI=(sexuality) OR KP=(sexuality) OR

AB=(education) OR TI=(education) OR KP=(education) OR

AB=(ableism) OR TI=(ableism) OR KP=(ableism) OR

AB=(race) OR TI=(race) OR KP=(race) OR

AB=(racism) OR TI=(racism) OR KP=(racism) OR

AB=(politics) OR TI=(politics) OR KP=(politics) OR

AB=(capitalism) OR TI=(capitalism) OR KP=(capitalism) OR

AB=(discrimination) OR TI=(discrimination) OR KP=(discrimination) OR

AB=(socioeconomic status) OR TI=(socioeconomic status) OR KP=(socioeconomic status) OR

AB=(Residence Characteristics) OR TI=(Residence Characteristics) OR KP=(Residence Characteristics) OR AB=(marginalized) OR TI=(marginalized) OR KP=(marginalized) OR

AB=(caste) OR TI=(caste) OR KP=(caste) OR

AB=(social class) OR TI=(social class) OR KP=(social class) OR

AB=(class background) OR TI=(class background) OR KP=(class background) OR

AB=(refugee) OR TI=(refugee) OR KP=(refugee) OR

AB=(religion) OR TI=(religion) OR KP=(religion) OR

AB=(homophobia) OR TI=(homophobia) OR KP=(homophobia) OR

AB=(ageism) OR TI=(ageism) OR KP=(ageism) OR

AB=(transphobia) OR TI=(transphobia) OR KP=(transphobia) OR

AB=(ethnocentrism) OR TI=(ethnocentrism) OR KP=(ethnocentrism) OR

AB=(heterosexism) OR TI=(heterosexism) OR KP=(heterosexism) OR

AB=(sexism) OR TI=(sexism) OR KP=(sexism) OR

AB=(war) OR TI=(war) OR KP=(war) OR

AB=(immigration) OR TI=(immigration) OR KP=(immigration) OR

AB=(economy) OR TI=(economy) OR KP=(economy) OR

AB=(classism) OR TI=(classism) OR KP=(classism))) **AND ((**AB=(Drug Resistance, Microbial) OR TI=(Drug Resistance, Microbial) OR KP=(Drug Resistance, Microbial) OR AB=(Drug Resistances, Microbial) OR TI=(Drug Resistances, Microbial) OR KP=(Drug Resistances, Microbial) OR

AB=(Antimicrobial Drug Resistance) OR TI=(Antimicrobial Drug Resistance) OR KP=(Antimicrobial Drug Resistance) OR

AB=(Antimicrobial Drug Resistances) OR TI=(Antimicrobial Drug Resistances) OR KP=(Antimicrobial Drug Resistances) OR

AB=(Antimicrobial Resistance, Drug) OR TI=(Antimicrobial Resistance, Drug) OR KP=(Antimicrobial Resistance, Drug) OR

AB=(Antimicrobial Resistances, Drug) OR TI=(Antimicrobial Resistances, Drug) OR KP=(Antimicrobial Resistances, Drug) OR

AB=(Drug Antimicrobial Resistance) OR TI=(Drug Antimicrobial Resistance) OR KP=(Drug Antimicrobial Resistance) OR

AB=(Drug Antimicrobial Resistances) OR TI=(Drug Antimicrobial Resistances) OR KP=(Drug Antimicrobial Resistances) OR

AB=(Resistance, Drug Antimicrobial) OR TI=(Resistance, Drug Antimicrobial) OR KP=(Resistance, Drug Antimicrobial) OR

AB=(Resistances, Drug Antimicrobial) OR TI=(Resistances, Drug Antimicrobial) OR KP=(Resistances, Drug Antimicrobial) OR

AB=(Antibiotic Resistance, Microbial) OR TI=(Antibiotic Resistance, Microbial) OR KP=(Antibiotic Resistance, Microbial) OR

AB=(Antibiotic Resistance) OR TI=(Antibiotic Resistance) OR KP=(Antibiotic Resistance) OR

AB=(Resistance, Antibiotic) OR TI=(Resistance, Antibiotic) OR KP=(Resistance, Antibiotic) OR

ALL=(Antibiotic Resistance) OR ALL=(Resistance))))

Cochrane library:

#1 MeSH descriptor: [Tuberculosis] explode all trees and with qualifier(s): [economics - EC, microbiology - MI, drug therapy - DT, epidemiology - EP, prevention & control - PC, genetics - GE, ethnology - EH, complications - CO, etiology - ET, diagnosis - DI]

#2 MeSH descriptor: [Malaria] explode all trees and with qualifier(s): [economics - EC, microbiology - MI, drug therapy - DT, epidemiology - EP, prevention & control - PC, genetics - GE, ethnology - EH, complications - CO, etiology - ET, diagnosis - DI]

#3 MeSH descriptor: [Neglected Diseases] explode all trees and with qualifier(s): [economics - EC, microbiology - MI, drug therapy - DT, epidemiology - EP, prevention & control - PC, genetics - GE, ethnology - EH, complications - CO, etiology - ET, diagnosis - DI]

#4 MeSH descriptor: [Leprosy] explode all trees and with qualifier(s): [economics - EC, microbiology - MI, drug therapy - DT, epidemiology - EP, prevention & control - PC, genetics - GE, ethnology - EH, complications - CO, etiology - ET, diagnosis - DI]

#5 MeSH descriptor: [Buruli Ulcer] explode all trees and with qualifier(s): [economics - EC, microbiology - MI, drug therapy - DT, epidemiology - EP, prevention & control - PC, genetics - GE, ethnology - EH, complications - CO, etiology - ET, diagnosis - DI]

#6 MeSH descriptor: [Chagas Disease] explode all trees and with qualifier(s): [economics - EC, microbiology - MI, drug therapy - DT, epidemiology - EP, prevention & control - PC, genetics - GE, ethnology - EH, complications - CO, etiology - ET, diagnosis - DI]

#7 MeSH descriptor: [Dengue] explode all trees and with qualifier(s): [economics - EC, microbiology - MI, drug therapy - DT, epidemiology - EP, prevention & control - PC, genetics - GE, ethnology - EH, complications - CO, etiology - ET, diagnosis - DI]

#8 MeSH descriptor: [Chikungunya Fever] explode all trees and with qualifier(s): [economics - EC, microbiology - MI, drug therapy - DT, epidemiology - EP, prevention & control - PC, genetics - GE, ethnology - EH, complications - CO, etiology - ET, diagnosis - DI]

#9 MeSH descriptor: [Dracunculiasis] explode all trees and with qualifier(s): [economics - EC, microbiology - MI, drug therapy - DT, epidemiology - EP, prevention & control - PC, genetics - GE, ethnology - EH, complications - CO, etiology - ET, diagnosis - DI]

#10 MeSH descriptor: [Echinococcosis] explode all trees and with qualifier(s): [economics - EC, microbiology - MI, drug therapy - DT, epidemiology - EP, prevention & control - PC, genetics - GE, ethnology - EH, complications - CO, etiology - ET, diagnosis - DI]

#11 MeSH descriptor: [Leishmaniasis] explode all trees and with qualifier(s): [economics - EC, microbiology - MI, drug therapy - DT, epidemiology - EP, prevention & control - PC, genetics - GE, ethnology - EH, complications - CO, etiology - ET, diagnosis - DI]

#12 MeSH descriptor: [Elephantiasis, Filarial] explode all trees and with qualifier(s): [economics - EC, microbiology - MI, drug therapy - DT, epidemiology - EP, prevention & control - PC, genetics - GE, ethnology - EH, complications - CO, etiology - ET, diagnosis - DI]

#13 MeSH descriptor: [Mycetoma] explode all trees and with qualifier(s): [economics - EC, microbiology - MI, drug therapy - DT, epidemiology - EP, prevention & control - PC, genetics - GE, ethnology - EH, complications - CO, etiology - ET, diagnosis - DI]

#14 MeSH descriptor: [Onchocerciasis] explode all trees and with qualifier(s): [economics - EC, microbiology - MI, drug therapy - DT, epidemiology - EP, prevention & control - PC, genetics - GE, ethnology - EH, complications - CO, etiology - ET, diagnosis - DI]

#15 MeSH descriptor: [Rabies] explode all trees and with qualifier(s): [economics - EC, microbiology - MI, drug therapy - DT, epidemiology - EP, prevention & control - PC, genetics - GE, ethnology - EH, complications - CO, etiology - ET, diagnosis - DI]

#16 MeSH descriptor: [Scabies] explode all trees and with qualifier(s): [economics - EC, microbiology - MI, drug therapy - DT, epidemiology - EP, prevention & control - PC, genetics - GE, ethnology - EH, complications - CO, etiology - ET, diagnosis - DI]

#17 MeSH descriptor: [Schistosomiasis] explode all trees and with qualifier(s): [economics - EC, microbiology - MI, drug therapy - DT, epidemiology - EP, prevention & control - PC, genetics - GE, ethnology - EH, complications - CO, etiology - ET, diagnosis - DI]

#18 MeSH descriptor: [Snake Bites] explode all trees and with qualifier(s): [economics - EC, microbiology - MI, drug therapy - DT, epidemiology - EP, prevention & control - PC, genetics - GE, ethnology - EH, complications - CO, etiology - ET, diagnosis - DI]

#19 MeSH descriptor: [Taeniasis] explode all trees and with qualifier(s): [economics - EC, microbiology - MI, drug therapy - DT, epidemiology - EP, prevention & control - PC, genetics - GE, ethnology - EH, complications - CO, etiology - ET, diagnosis - DI]

#20 MeSH descriptor: [Cysticercosis] explode all trees and with qualifier(s): [economics - EC, microbiology - MI, drug therapy - DT, epidemiology - EP, prevention & control - PC, genetics - GE, ethnology - EH, complications - CO, etiology - ET, diagnosis - DI]

#21 MeSH descriptor: [Trachoma] explode all trees and with qualifier(s): [economics - EC, microbiology - MI, drug therapy - DT, epidemiology - EP, prevention & control - PC, genetics - GE, ethnology - EH, complications - CO, etiology - ET, diagnosis - DI]

#22 MeSH descriptor: [Yaws] explode all trees and with qualifier(s): [economics - EC, microbiology - MI, drug therapy - DT, epidemiology - EP, prevention & control - PC, genetics - GE, ethnology - EH, complications - CO, etiology - ET, diagnosis - DI]

#23 ("human African trypanosomiasis"):ti,ab,kw (Word variations have been searched)

#24 ("soil transmitted helminth"):ti,ab,kw (Word variations have been searched)

#25 ("ectoparasite"):ti,ab,kw (Word variations have been searched)

#26 (foodborne trematod*):ti,ab,kw

#27 (Tuberculosis):ti,ab,kw

#28 (Malaria):ti,ab,kw

#29 (leprosy):ti,ab,kw

#30 (Buruli ulcer):ti,ab,kw

#31 ("Chagas disease"):ti,ab,kw

#32 (dengue):ti,ab,kw

#33 (chikungunya):ti,ab,kw

#34 (dracunculiasis):ti,ab,kw

#35 (echinococcosis):ti,ab,kw

#36 (leishmaniasis):ti,ab,kw

#37 (elephantiasis filarial):ti,ab,kw

#38 (lymphatic filariasis):ti,ab,kw

#39 (mycetoma):ti,ab,kw

#40 (chromoblastomycosis):ti,ab,kw

#41 (deep mycoses):ti,ab,kw

#42 (onchocerciasis):ti,ab,kw

#43 (taeniasis):ti,ab,kw

#44 (cysticercosis):ti,ab,kw

#45 (trachoma):ti,ab,kw

#46 ("snake bites"):ti,ab,kw (Word variations have been searched)

#47 (yaws):ti,ab,kw (Word variations have been searched)

#48 (schistosomiasis):ti,ab,kw (Word variations have been searched)

#49 (rabies):ti,ab,kw (Word variations have been searched)

#50 (scabies):ti,ab,kw (Word variations have been searched)

#51 (neglected tropical disease):ti,ab,kw (Word variations have been searched)

#52 (noma):ti,ab,kw (Word variations have been searched)

#53 #1 OR #2 OR #3 OR #4 OR #5 OR #6 OR #7 OR #8 OR #9 OR #10 OR #11 OR #12 OR #13 OR #14 OR #15 OR #16 OR #17 OR #18 OR #19 OR #20 OR #21 OR #22 OR #23 OR #24 OR #25 OR #26 OR #27 OR #28 OR #29 OR #30 OR #31 OR #32 OR #33 OR #34 OR #35 OR #36 OR #37 OR #38 OR #39 OR #40 OR #41 OR #42 OR #43 OR #44 OR #45 OR #46 OR #47 OR #48 OR #49 OR #50 OR #51 OR #52

#54 MeSH descriptor: [Sexism] explode all trees

#55 MeSH descriptor: [Gender-Affirming Care] explode all trees

#56 MeSH descriptor: [Gender Identity] explode all trees

#57 MeSH descriptor: [Social Class] explode all trees

#58 ("gender identity"):ti,ab,kw (Word variations have been searched)

#59 (sex):ti,ab,kw (Word variations have been searched)

#60 (discrimination):ti,ab,kw (Word variations have been searched)

#61 (sexuality):ti,ab,kw (Word variations have been searched)

#62 (caste):ti,ab,kw (Word variations have been searched)

#63 (social class):ti,ab,kw (Word variations have been searched)

#64 (class background):ti,ab,kw (Word variations have been searched)

#65 (refugee):ti,ab,kw (Word variations have been searched)

#66 (religion):ti,ab,kw (Word variations have been searched)

#67 (ageism):ti,ab,kw (Word variations have been searched)

#68 (transphobia):ti,ab,kw (Word variations have been searched)

#69 (ethnocentrism):ti,ab,kw (Word variations have been searched)

#70 (heterosexism):ti,ab,kw (Word variations have been searched)

#71 (immigration):ti,ab,kw (Word variations have been searched)

#72 (classism):ti,ab,kw (Word variations have been searched)

#73 (war):ti,ab,kw (Word variations have been searched)

#74 (gender expression):ti,ab,kw (Word variations have been searched)

#75 ("gender-associated"):ti,ab,kw (Word variations have been searched)

#76 (sexism):ti,ab,kw (Word variations have been searched)

#77 (disability):ti,ab,kw (Word variations have been searched)

#78 (ethnicity):ti,ab,kw (Word variations have been searched)

#79 (capitalism):ti,ab,kw (Word variations have been searched)

#80 (gender orientation):ti,ab,kw (Word variations have been searched)

#81 (sex orientation):ti,ab,kw (Word variations have been searched)

#82 (elder):ti,ab,kw (Word variations have been searched)

#83 (marginalized):ti,ab,kw (Word variations have been searched)

#84 #54 OR #55 OR #56 OR #57 OR #58 OR #59 OR #60 OR #61 OR #62 OR #63 OR #64 OR #65 OR #66 OR #67 OR #68 OR #69 OR #70 OR #71 OR #72 OR #73 OR #74 OR #75 OR #76 OR #77 OR #78 OR #79 OR #80 OR #81 OR #82 OR #83

#85 MeSH descriptor: [Drug Resistance, Microbial] explode all trees

#86 (Antimicrobial Drug Resistance):ti,ab,kw (Word variations have been searched)

#87 (multi drug resistance):ti,ab,kw (Word variations have been searched)

#88 (poly drug resistance):ti,ab,kw (Word variations have been searched)

#89 ("drug-resistance"):ti,ab,kw (Word variations have been searched)

#90 (antibiotic resistance):ti,ab,kw (Word variations have been searched)

#91 #85 OR #86 OR #87 OR #88 OR #89 OR #90

#92 #53 AND #84 AND #91
